# Smallpox Geographies: vaccination, borders and Indigenous peoples in Australia’s coastal north

**DOI:** 10.1017/mdh.2023.39

**Published:** 2024-01

**Authors:** Chi Chi Huang, Alison Bashford

**Affiliations:** Department of Humanities and Languages, UNSW, Sydney, Australia

**Keywords:** Vaccination, Smallpox, Indigenous Peoples, Australia in Southeast Asia, Medical Geography, Coastal Borders

## Abstract

Australia’s approach to its biosecurity and borders has always been two-pronged – quarantine first, vaccination second. This article asks what this combination looked like in practice by exploring two neglected smallpox vaccination campaigns directed towards Indigenous peoples in the early twentieth century. We argue these were important campaigns because they were the first two pre-emptive, rather than reactionary, vaccination programs directed towards First Nations people. Second, both episodes occurred in Australia’s northern coastline, where the porous maritime geography and proximity to Southeast Asia posed a point of vulnerability for Australian health officials. While smallpox was never endemic, (though epidemic), in Australia, it was endemic at various times and places across Southeast Asia. This shifting spectre of smallpox along the northern coastline was made even more acute for state and federal health officials because of the existing polyethnic relationships, communities, and economies. By vaccinating Indigenous peoples in this smallpox geography, they were envisioned and embedded into a ‘hygienic’ border for the protection of white Australia, entwining the two-prongs as one approach. In this article, we place public health into a recent scholarship that has ‘turned the map upside down’ to re-spatialise Australia’s history and geography to the north and its global connections, while demonstrating how particular coastlines and their connections were drawn into a national imaginary through a health lens.

## Introduction

Australia is known internationally for its stringent quarantine and biosecurity measures. Even before the COVID-19 pandemic, the nation’s strict border policies had been the subject of amusement in Western popular culture, even satirised in *The Simpsons.* ^1^ It was no surprise that in responding to the SARs-CoV-2 in 2020, Australia closed its international border entirely, prompting media outlets to both praise and criticise the emergence of a ‘Fortress Australia’.^2^ Despite oscillating opinions, it was a novelty, a peculiarity, to see a Western democratic nation ‘draw up its bridges’ so suddenly.^3^ Those who were not citizens or permanent residents were barred from entering. Returning residents who were lucky enough to win a seat on a government flight or who were financially able to purchase a plane ticket were escorted by military personnel into two-week long hotel quarantines at their own expense. Later, other citizens had to remain in their international locations, refused entry to their own homeland, prompting legal questions about ‘the right to return’.^4^ Citizens within this ‘hermit kingdom’ were also prevented from leaving, a policy that ‘not even China’, exclaimed *Foreign Policy*, had implemented.^5^ These restrictions were in place, citizens were reminded, until Australia could ‘arm’ itself with a viable vaccine.^6^

This two-pronged approach—quarantine and border control first, vaccination second—has long been envisioned as the two lines of defence in Australia’s approach to the management of infectious diseases. Writing in 1909, Dr J.H.L Cumpston, a leading figure who shaped Australia’s public health apparatuses, stated that ‘hygienists in Australia will look on the seaward frontiers as the places which must be fully manned and equipped with the most modern armamentaria in order that the possibility of invasion by disease shall be reduced to an absolute minimum’.^7^ The network of quarantine facilities along the coast were alert and ready to act: spaces of inspection, isolation, and fumigation that would keep the island nation free from microbial threats. Cumpston’s statement echoed earlier sentiments expressed in the late nineteenth century to develop and standardise quarantine measures and regulations across the then six self-governing colonies.^8^ These conversations took place in parallel with debates about federation of those (British) colonies into a new nation-state. When New South Wales, Victoria, Tasmania, Queensland, South Australia, and Western Australia became the Commonwealth of Australia in 1901, public health remained under the remit of each new state, but powers of quarantine fell under federal administration, and this enabled, as argued by Alison Bashford, a ‘particular geographic imagining’ of the nation that drew attention to its boundaries and territoriality.^9^ Quarantine was paramount in understanding the health of the country, but as Cumpston noted, ‘vaccination should be continued as a preventative measure’.^10^ This medical procedure complemented quarantine and was envisioned as the country’s second line of defence against smallpox. The twinning of these two health technologies has been noted by scholars, but this article asks, what did the pairing of quarantine and vaccination look like in practice?

To answer this question, this article explores Australia’s smallpox vaccination history with a focus on Indigenous communities in the early twentieth century. The few histories of vaccination in Australia have focused either on the nineteenth century, or on the post-World War Two period, and have neglected two smallpox vaccination campaigns, one in 1912 and the second in 1933, which were directed towards the provision of Indigenous peoples.^11^ Yet these were important campaigns: they were the first two pre-emptive, rather than reactionary, vaccination programs directed towards First Nations people. The campaign in 1912 vaccinated 1279 Torres Strait Islanders as part of a health surveillance tour overseen by the new Queensland Commissioner of Public Health, Dr J.S.C. Elkington.^12^ In 1933, Dr Cecil Cook, Chief Protector of Aborigines and Chief Health Officer of the Northern Territory, vaccinated 272 Indigenous people in Darwin and on Bathurst Island after seeking federal approval in consultation with Australia’s Director-General of Health and Director of Quarantine, Dr J.H.L. Cumpston.^13^ In both instances, the immediate threat of smallpox to Australia’s onshore population was low. There were no exceptional outbreaks within the country or in neighbouring territories to cause excess concern about the disease seeping through Australia’s quarantine and breaching its borders. However, in the minds of these health officials, it was prudent to undertake this prophylactic measure. This aligned with the already longstanding public health view of the Australian continent as relatively free from external disease threats: smallpox was indeed epidemic and never endemic in the island nation, and early twentieth-century health officials started to turn to prophylactic vaccination in regions and populations considered to be risky contact zones.

**Figure 1.**
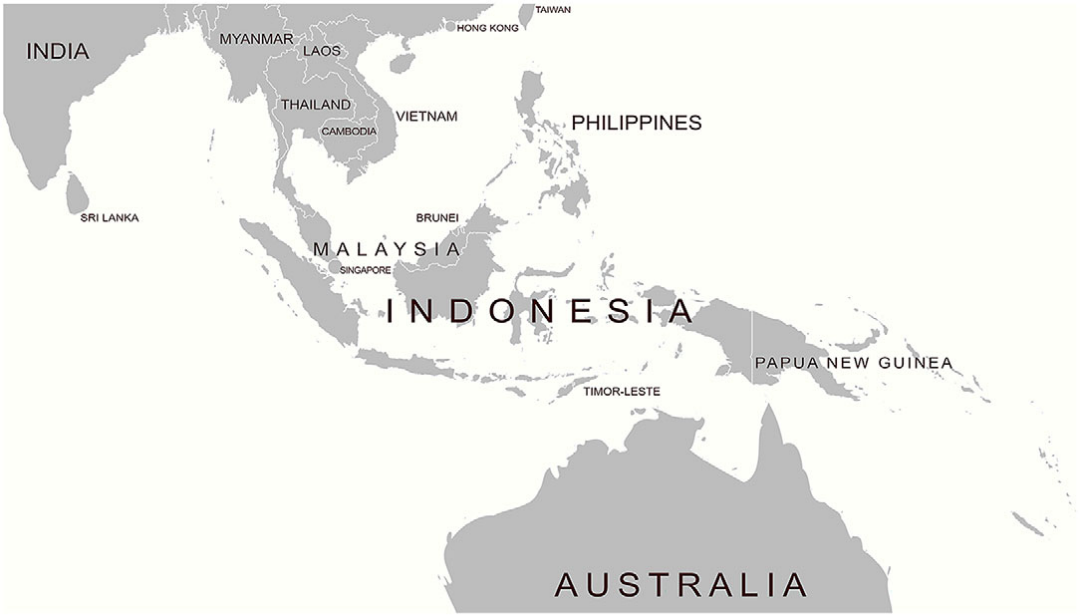
Map of Northern Australia and Southeast Asia, Source: Created by Chi Chi Huang, 2023.

Spaced two decades apart, this article analyses these two episodes together, arguing that vaccinated Aboriginal and Torres Strait Islanders became part of Australia’s first line of defense against smallpox in a porous northern maritime geography [Figure 1]. Throughout the nineteenth and early twentieth centuries, northern Australia, proximate to Southeast Asia, was understood as a frontier zone to which white administrators, explorers, business opportunists, and the public needed to bring order, as well as civic and economic cultivation.^14^ Pearling was an influential industry in northern Australia and the coastlines of Darwin and the Torres Strait were centres for economic activity that employed multi-racial and multi-ethnic labourers whose formal nationality might have been the Dutch East Indies, the British Straits Settlements, German New Guinea, or Portuguese Timor. The pearling industry was the only industry exempt from Australia’s race and health-based *Immigration Act 1901*, and it endured with the continuance of indentured labour until the 1970s.^15^ Yet from a quarantine and public health point of view, pearlers’ mobility across the waters between Southeast Asia and northern Australia were identified as a point of vulnerability: quarantine was feared to be insufficient.

A recent school of historians has explored this industry and region closely, calling for scholars to ‘turn the map upside down’.^16^ This re-spatialises Australia’s geography and history, disrupting the traditional British Empire focus on the Pacific south-east that has long privileged James Cook’s ‘first contact’ (1770) and the British ‘first fleet’ of convicts (1788) sent to Botany Bay, as an original place and time for the nation. Centuries of Macassan trading contact across the Torres Strait, for example, now vies with conventional British history and historiography orientations, establishing a different historical geography of the continent, its Indigenous peoples, and contact with outsiders. Historians Lynette Russell, David Walker, Regina Ganter, Julia Martinez, Adrian Vickers, and others have shown the lengthy economic and intimate social entanglements that the north has had with the Indonesian archipelago, South East Asia and East Asia, prior to and during British colonisation.^17^ Taken together these histories also show how white Australian anxieties about a poly-ethnic north led to policies at various governmental scales to regulate these social and economic connections.^18^ As a result, ‘Aboriginality’, as a category for governance, was defined as distinct and kept separate from ‘Asian’, denying the mixed relations that have historically existed along the coastal north of the continent.^19^ This article adds a public health component to the racial politics of northern Australia, which was key to the construction of the nation’s identity as relatively disease-free, as ‘hygienic’.

This scholarship, which geographically re-orients the nation’s history, speaks directly to the significance of coasts and maritime quarantining of the island continent; sites for distilled activity, concern, anxiety, and exchange. The imagining of the island nation has been explored by Bashford in the health context and Frank Broeze in the cultural and political context.^20^ They show the multifarious ways in which Australia has been geographically and ideologically bounded as an island of social, political, and cultural whiteness, as an ‘antipodes’ in Asia, and fragmented from Britain. Indeed, the history of quarantine in the geo-political construction of the nation has been a large component in this social imaginary. Yet these histories also tend to locate the centre of these ideologies and power in the south-east of the continent, whence concerted efforts of state-making and territorial expansion emanated, and where administrative decision-making took place: the federal capital was located first in Melbourne and then Canberra.^21^ There is a need for Australian public health histories to also move to the coastal north, and in doing so also move beyond the historiographical interest in ‘the tropics’ and ‘tropical hygiene’.^22^

The decision to target Aboriginal and Torres Strait Islanders specifically in 1912 and 1933 requires explanation and brings to the fore the place and function of Indigenous health for an emerging settler-colonial nation. After an initial outbreak in 1789 that immediately followed the arrival of British ships on Gadigal land, there were intermittent outbreaks across the nineteenth and early twentieth centuries, though smallpox was never endemic. However, that very fact also made the whole continent’s population more vulnerable. Its herd immunity from the disease itself was not strong. Thus, by the early twentieth century, it was a sensible-enough idea, in public health terms, to pre-emptively vaccinate populations on the porous northern coastline, proximate to places where smallpox *was* endemic and had long been so. Most of the medical establishment in Australia advocated for a greater proportion of the total population to take up vaccinations against smallpox that were generally safe, even routine, although the death in a Queensland town of twelve children from infected diphtheria toxin-antitoxin in 1928 certainly compromised public health public relations.^23^

White Australians generally could choose whether or not to have themselves or their children vaccinated. In the 1912 and 1933 vaccination campaigns examined here, Aboriginal and Torres Strait Islanders could not. Their bodies were incorporated into a northern frontier zone of defence, and their prophylactic immunity conceptualised as a border against disease transmission into the Australian continent and population. To explore the significance of these campaigns and how all these questions were intertwined, this article will first establish the hold that smallpox had on Australian administrators in thinking about the biosecurity of the colonies and nation. A discussion of the extent to which Australia was vaccinated against the disease will follow, contextualizing a closer examination of the episodes in 1912 and 1933. Regulating the smallpox geographies of the polyethnic coastal north, proximate to South East Asia, was part of the production of ‘white’ Australia.

## The Spectre of Smallpox

Smallpox holds an important place in the medical history of colonialism and of many settler nations.^24^ In the Australian historical and historiographical context, the smallpox story mirrors the north versus south-east historical (and historiographical) geography. One thesis argues that smallpox pre-existed British colonisation in 1788, interestingly—given our current geographical interest—suggesting that it had entered with Macassan traders in the ‘monsoonal north’ in the mid-eighteenth century.^25^ Most historical and epidemiological researchers, however, still understand First Nations people on the Australian continent to have had no prior exposure to smallpox, explaining the great tragedy of mortality and morbidity beginning in April 1789, the year after the British arrived in Sydney Cove, in the south-eastern coast of the continent.^26^

The history of smallpox in Australia is peculiar largely because of its minimal significance for the white population and its disproportionate significance in Indigenous history.^27^ After the great dying of 1789, other smallpox outbreaks affecting First Nations communities were recorded in the 1830s and the 1860s. From the 1870s onwards, there were recorded observations of the characteristic scars amongst some Indigenous people in the north.^28^ Pointedly excluding these Indigenous cases, J.H.L. Cumpston noted just 2962 instances of smallpox across Australia between 1857 and 1921.^29^ Just over one thousand of these appeared during a 1913 epidemic in Sydney.^30^ Smallpox was not prevalent in Australia and certainly not endemic. It was, rather, a disease of the old world, or as Elkington put in 1912, a ‘terrible scourge from which Australia alone amongst the continents of the world is free’.^31^ But, he cautioned, smallpox ‘is always knocking at our doors’.^32^ The fear of the disease was exacerbated in the mid to late nineteenth century by accelerating modes of travel and by increasing global trade, bringing Australia closer in time and space to any potential infection. In 1868, Francis Campbell, a leading vaccinator in New South Wales, cautioned that ‘as commerce with all quarters of the globe and immigration from all countries increase, so will the probability of the introduction of small-pox increase in proportion’.^33^ And, a ‘consequence of the substitution of steamships for sailing vessels’, noted Haynes Gibbes Alleyne, a leading health officer in the colony of New South Wales, in 1878, would be that ‘year by year our intercourse with countries where small-pox always prevail…becomes more frequent and rapid’.^34^ It was precisely its absence that kept health administrators vigilant and concerned about the perpetual threat of smallpox.

On the few occasions that an epidemic of the disease was declared, the panic and reactions affected public health systems across Australia. Peter Curson details how the crisis and chaos of the 1881 epidemic in Sydney led to the creation of the New South Wales (NSW) Health Board and other foundational public health structures for the colony.^35^ Looking at the same epidemic, Bashford examines how the issue of consent came to feature in thinking about public health.^36^ And although the meaning of consent varied across different demographics, there was a shift towards managing population health in increasingly governmental modes of education, persuasion, and self-responsibility.^37^ The success of administrative changes was shown to be inconsistent by Michael Roe who argued that the reforms generated by an 1887 epidemic in Launceston, Tasmania, had no lasting effect, as the city was similarly unprepared and panicked when smallpox struck again in 1903.^38^ Public health responses and discussion about more stringent vaccination policies were typically reactive to intermittent epidemics, not proactive, longer-term schemes to build population immunity.

Attempts to manage the disease and prepare for potential smallpox visitations also served to distinguish Australia as independent and unique. In the late nineteenth century, at a time when Britain, domestically, was moving away from quarantine as the preferred method of disease management, the Australian colonies continued to develop ever-more stringent border policies and procedures.^39^ The disagreement on the treatment of smallpox cases on board the *HMS Wolverene*, SS *Brisbane*, and the Pacific Mail Steamship *Australia* between 1876 and 1877 showcased ‘different scales of sovereignty’ being exerted by the colonial government of New South Wales and the limits of imperial sovereignty, as Peter Hobbins explores.^40^ Simultaneously, ‘Australia’s perceived threat from the seas’ states Hobbins, ‘shifted decisively towards East Asia’ around the 1880s.^41^ So when smallpox re-appeared in 1881 after the triple vessel occurrence of the 1870s, there was no doubt in the Australian public discourse that the disease originated from a Chinese boy on board, despite reassurances from medical professionals that ‘there was absolutely no evidence of the origin’ attributed to that child.^42^ By virtue of being Chinese, the press made an immediate link between him and the recent quarantine of the SS *Brisbane*, carrying some 350 Chinese men from Hong Kong.^43^ The racial vilification of migrant groups during outbreaks of disease worldwide is well-established, and this turn towards comprehending nearby East and South East Asia as the source of disease intensified in the late nineteenth century.^44^ The increasing insistence on the Asiatic origins of diseases fuelled the belief that Australia, by virtue of its geography, was under an ever-present threat of being overwhelmed by people and microbes from China in particular, and East Asia in general. What this sequence of smallpox outbreaks shows is how the particular disease of smallpox became cemented as a discursive entity in the formation of identity politics in the lead up to the federation of Australia in 1901. It happened, also, to be the singular disease for which a vaccine had long been available.

## Australia’s ‘unvaccinated condition’

The commitment to quarantine and disease surveillance of borders was also a result of Australia’s largely ‘unvaccinated condition’. In 1796, Edward Jenner was the first to demonstrate that vaccination—the use of cowpox (vaccinia virus) to inoculate individuals against smallpox (variola virus)—was effective in mitigating the spread and severity of the disease. The practice was quickly adopted across the world, and, by the end of the nineteenth century, vaccinations had reduced the impact of smallpox in Europe and North America. Compulsory vaccination laws were also used to compel populations to take up the procedure.^45^ The *Vaccination Act* 1853 in Britain required infants to be vaccinated within the first three months of their life, and this served as a model for similar mandates in the Australian colonies. In that same year, South Australia passed the first Vaccination Act, with Victoria and Tasmania following suit in 1854, and Western Australia in 1860. Although attempts were made in New South Wales, the colony that Cumpston noted ‘has suffered most from small-pox’, there was never any legislation passed or a coordinated scheme devised for mandatory smallpox vaccinations.^46^ Queensland’s *Health Act* 1900–1922 articulated compulsory vaccination, but this part of the Act was never proclaimed, rendering the procedure entirely voluntarily.^47^ The fact that this measure of the Act had not been brought into operation was commented upon in Queensland newspapers in 1911, when a single case of smallpox arrived in Sydney in September that year.^48^ In such moments of heightened awareness, debates around compulsory smallpox vaccination were routinely revisited with proponents of preventative action alert to the potential of a public health disaster.

The existence of compulsory vaccination laws did not result in a strongly vaccinated population. The Principal Medical Officer of Western Australia admitted that in the early 1900s ‘the Vaccination Act was practically ignored’.^49^ While Tasmania had its Act in place by 1854, vaccinations were ‘entirely suspended in the State’ between 1868–1877 and only resumed in late 1877 because there was a rush for protection after an outbreak in Sydney.^50^ By 1910, universal vaccination in Australia was ‘so incomplete that it cannot have opposed any effective barrier against the establishment of the disease in endemic or even in epidemic form,’ concluded Cumpston.^51^ Taking into account the variability and availability of each States’ records, ‘it is fairly safe to say’, reported Cumpston, ‘that 30 percent of the Australian population has at one time been vaccinated’.^52^ Furthermore, the introduction of various conscientious objections, as detailed in Table 1, rendered many of these compulsory vaccination acts essentially voluntary in nature. While legislation existed in most colonies and states, they were not implemented with any rigour.

**Table 1.** Vaccination legislation in Australian colonies, states, and the Commonwealth of Australia, 1853–1925 Compiled from: J.H.L. Cumpston, *The History of small-pox in Australia, 1788 – 1908*, (Melbourne, Government Printer, 1914),130-46; J.H.L. Cumpston and F. McCallum, *The History of Small-pox in Australia 1909-1923*, (Melbourne: H.J. Green, Government Printer, 1925), 98–108; J.H.L Cumpston, introduced and edited by M.J. Lewis, *Health and Disease in Australia: A History*, (Canberra: Australian Government Publishing Service, 1989), 188–200; Quarantine Act 1908 (Cth).

New South Wales		
None was ever passed. There was an attempt to pass legislation in 1853, 1869, 1877, and 1913.		

Victoria		
1854	An Act to make the Practice of Vaccination compulsory	Every infant was required to be vaccinated within the first three months after birth.
1865	Public Health Statute of 1865, Part V.	The previous act was subsumed under the Public Health Act of 1865.
		Every infant was required to be vaccinated within the first six months after birth.
1874	Vaccination Act 1874	The vaccination requirements in the Public Health Act of 1865 was replaced by this Act.
1890	Consolidated Health Act	Previous Act was replaced by this Act, Part IX.
1919	Health Act of 1919	Incorporated a conscience objection clause, which came into force in 1920.

Queensland		
1900	Health Act of 1900	Provided for compulsory vaccination but never proclaimed; thus, no legislation existed to enforce and apply this procedure.

South Australia		
1853	‘Vaccination Act’ Act no. 16 of 1853	Every child to be vaccinated within four to six months after birth, depending on distance to public vaccinator.
1854	Act No. 15 of 1854	Valid vaccination certificate required to prove that newborns had been vaccinated.
1872	Act No, 21 of 1872	Every newborn to be vaccinated within six months after birth, except in cases where the Public Vaccinator deemed the child to be unfit or insusceptible to the procedure.
		Required inspection of vaccination success after seven days.
1882	Vaccination Act 1892	Repealed all previous acts.
		Compulsory vaccination of newborns within the first six months after birth, except in cases of unfitness or insusceptibility.
		Police empowered to take action, if required.
		Inoculation with smallpox virus was prohibited, though vaccination officers were required to keep both animal and humanized lymphs in stock.
		Empowered the Governor to declare an area as infected and thus enforce other regulations that may be necessary for the greater public.
1892	Act No. 554 of 1892	Gave parents the option to request calf or humanised lymph for the procedure on their newborn within the first six months of birth.
1901	An Act to Abolish Compulsory Vaccination	Exemption from penalty, provided the parents made a declaration of conscience objection within the first six months of newborn’s birth, and a letter must be delivered to a Vaccination Officer within seven days of declaration.
		Government was empowered to override this act in the occurrence of smallpox within the community and compel any contact to be vaccinated or re-vaccinated.
		Various amendments in 1906 and 1911 extended the enforcement of this Act until 1916.
1917	Compulsory Vaccination Exemption Act	Suspended compulsory vaccination of children.

Western Australia		
1860	An Ordinance to make Compulsory the Practice of Vaccination	Each child residing within ten miles of a District Vaccinator had to be vaccinated within three months of birth.
		Each child residing outside of ten-mile radius was to be brought to the District Vaccinator for procedure within six months.
		Inoculation with smallpox virus was prohibited.
1878	Vaccination Act	Each child residing within five miles of a District Vaccinator had to be vaccinated within three months of birth.
		Each child residing outside of twenty-mile radius was to be brought to the District Vaccinator for the procedure within six months.
		All newborns residing outside the twenty-mile radius was to be vaccinated by a travelling Public Vaccinator.
	Health Act, 1911	A conscience objection clause was inserted and very few vaccinations were performed thereafter.

Tasmania		
1854	Vaccination Act	
1868-77	Vaccinations were suspended.	
1881	Vaccination Act	Replaced and repealed previous Act.
		Every person who had not been successfully vaccinated must submit themselves for the procedure within two months of the passing of this Act.
		Every newborn was to be vaccinated within two months after birth.
		
1882		Every newborn was to be vaccinated within six months after birth.
1890–7	Vaccinations were suspended.	
1898		Every newborn was to be vaccinated within twelve months after birth.
1903	Public Health Act	All sections pertaining to compulsory vaccination were taken out. No active legislation.

Commonwealth of Australia		
	Quarantine Act 1908 (Cth)	Under clause 75.1, a quarantine Officer may require any person subject to quarantine or performing quarantine duties to be vaccinated.

Colonial and state mandates in Australia were not sufficient to persuade people to get themselves or their children vaccinated, but outbreaks were. Between 1850 and 1914, increases in vaccinations amongst the white population typically occurred during an outbreak or in the immediate aftermath of a smallpox occurrence.^53^ Unsurprisingly, the greatest surge in vaccinations occurred in response to the largest smallpox outbreak in Australia, in Sydney, 1913. Given the spread of infections, quarantine boundaries were placed around Sydney, and vaccinations were a requirement to exit the medical border. In 1913, the rate of vaccination in New South Wales was 19.5 per 100 births, and in 1914, it was 12.4. This was a significant increase from 0.02 to 0.7, the average rate since records were kept until 1925.^54^

Similarly, it was also in moments of observed smallpox presence that calls were made to vaccinate Indigenous people, with the earliest record around 1830 in New South Wales. Dr John Mair noticed the tell-tale marks of the disease upon his arrival to the colony in 1827. After procuring fresh lymph in 1830, he was able to vaccinate some First Nations people in Bathurst, an inland town, who appeared ‘much afflicted with the smallpox’.^55^ Officials in Adelaide had early observed pockmarks within the Indigenous community compelling George Galwer, the Governor of South Australia, to instruct that vaccinations be ‘performed for the Native Inhabitants, every Wednesday, from eleven to twelve am, at the Native Huts on the Torrens’.^56^ Historian Judy Campbell mentions that the ‘vaccination of Aboriginal people in the south-west of Western Australia had begun by the 1850s’, but these measures were also prompted by a reaction to observed smallpox in a new colony.^57^ Founded in 1829, European settlers of Western Australia had noted traces of smallpox on some Aboriginal people in the 1840s. Western Australia became a penal colony in 1849, bringing a population increase, as well as a fear that ‘new diseases…will follow them [convicts and settler] especially the smallpox’.^58^ Concerned individuals such as H. Burnham Bryan, a surgeon of Brusselton, and Emily Mary Withnell, an activist for Indigenous protection, took it upon themselves in the 1850s and 1860s, respectively, to vaccinate any First Nations people who presented themselves for the procedure.^59^ While W. Cowan, the colony’s Guardian for Aboriginals in 1852, directed medical officers in Perth and York to order lymphs and ‘to induce them [First Nations] to submit to vaccination’.^60^ Governor Captain Charles Fitzgerald only ‘arrange[d] a plan for the vaccination of the Aborigines throughout the colony’ in 1854 after the new Guardian of Aborigines, Charles Symmons, reported the spread of smallpox in New South Wales would lead to ‘its speedy propagation through the other Australian Colonies…and must inevitably entail on an aboriginal population’.^61^

In addition to reacting to pox marks on Indigenous bodies, administrators would endeavour to vaccinate Indigenous communities when smallpox was suspected in other non-white people. For example, in 1880s Palmerston, as the city of Darwin was called before 1912, there was a sudden increase in Chinese immigrants and several confirmed cases of smallpox amongst them. Although health administrators in the late nineteenth century believed the introduction of smallpox amongst the Indigenous population of the north of Australia had come through the Indonesian Archipelago, it was Chinese immigration that spurred P.M. Wood, Colonial Surgeon and Protector, to try to vaccinate ‘the native population of Palmerston, assuring their protection from smallpox introduced to the port from time to time by Chinese immigrants’.^62^ When three cases of smallpox were confirmed in Broome, Western Australia, in 1903, compulsory vaccination and localised restriction on movement were applied to the pearling labourers and Indigenous people.^63^ Across the nineteenth-century colonies and early twentieth-century states, then, schemes to vaccinate First Nations people were reactive, prompted by a present or imminent outbreak of smallpox. The campaign to vaccinate Torres Strait Islanders in 1912 was different.

## 1912: Vaccinating Torres Strait Islanders under J.S.C. Elkington

J.S.C. Elkington assumed his post as Queensland’s commissioner of public health in January 1910. The Queensland *Annual Report of the Commissioner of Public Health* published 30 June that year identified smallpox as a disease of concern.^64^ While previous reports mentioned smallpox, they did so only in reference to the state’s key ports. The 1908 *Annual Report,* for example, logged cases of smallpox in three vessels prior to arriving in Brisbane. Brief details followed about the surveillance of infected vessels prior to docking, and when and where inspection, disinfection, quarantine, and release of people and the vessel took place.^65^ This was all routine. Once Elkington became commissioner, however, Queensland’s vulnerability to smallpox was prioritised. Within the first seven months of his post, Elkington designed and distributed ‘Inquiry and Detail Cards for Epidemic Disease (Smallpox)’ for official use.^66^ In describing the ‘geographical and epidemiological relationship of Queensland’, he warned that the connections between ‘Tropical Queensland with Eastern ports and countries are of much significance from the epidemiological standpoint’ because these places were ‘infected with smallpox, or have been recently so infected’.^67^

Elkington’s concerns were shared by Hon. J.G. Appel, the home secretary. On 28 April 1911, Appel embarked on a routine tour inspecting the management of missions and schools in the north of Queensland and the Torres Strait Islands.^68^ Although Appel was impressed by the conditions on the islands he visited (Badu, Mabuiag, Yam, Darnley, Murray, and Naghir), he still had ‘a number of questions relating to the physical health and welfare of the natives’ at the conclusion of his tour, and Elkington was the man to consult.^69^ Appel directed Elkington in July 1911 to ‘go thoroughly into…and report to him on certain epidemiological features of the Torres Strait Islands’.^70^ This report culminated in the 1911 *Annual Report,* widely discussed in the press. The ‘menace of the Torres Straits’, as one newspaper called it, ‘deserved attention, both from the standpoint of the preservation of the interesting races inhabiting them, and from that of the safety of Queensland’.^71^ Although Appel’s concern had been triggered by a bout of dysentery on the Islands, Elkington singled out smallpox.^72^ He observed that ‘smallpox has within recent years prevailed in these and other Asiatic dependencies…[i]t is not at all certain whether Dutch New Guinea is free from epidemic smallpox’.^73^ And if it was not free, then ‘it was likely to become epidemic at any time’.^74^ To Elkington, not only had the disease ‘prevailed’ by taking over much of South and Southeast Asia, the true extent of the spread seemed unknowable, and thus the neighbouring landmasses were on the ever-present verge of an outbreak. ‘The most imminent disease risk with which Queensland has to deal with’ he concluded, ‘is that of smallpox’.^75^

Pointedly, it was northern Queensland’s ‘geographical relationships’ with Asia that made the ‘advent’ of smallpox ‘to her shores a matter of practical certainty’.^76^ When he took the post, Elkington pushed to create a Northern Office of the Health Department based in Townsville, which opened in January 1912. The northern coast of the state posed a particular health problem, which could threaten not just the health of the rest of the state, but its coffers. He reasoned that at a ‘very low estimate’, a smallpox outbreak could cost Queensland from ‘£50,000 to £70,000’.^77^ If this financial burden was not concerning enough, Elkington illustrated his point about proximity by locating northern Queensland in time and distance to the threat:

A twelve-knot steamer could, after allowing for ordinary stoppages and detentions, land a smallpox-infected person from the Aru Islands, the Spice Islands, Celebes, Borneo, Manila, Timor, or Java, in either Cairns or Townsville, some two or more days before his illness began, or before there would be the smallest chance of its detection by a quarantine officer. An infected person from the Aru Islands could even be landed in Brisbane some days before the expiration of his incubation period. The first forty-eight hours of an ordinary attack of smallpox resemble a smart attack of dengue or influenza, and the characteristic eruption does not appear until the third day. Although the sufferer is highly infectious during the stage preceding the eruption, it is improbable that sufficient suspicion would be aroused to secure his effective isolation.^78^

Elkington hypothesised a temporal and spatial narrative of the variola virus transmission. This visualisation of how disease spread was aided by technological advancements, particularly aerial, which condensed and reconfigured Australia’s relationship to ‘microbial dangers’, as Bashford has argued, and provoked the Commonwealth Department of Health to rethink and adapt policies of migration and disease management and to join international networks of infectious disease surveillance.^79^ But for Elkington, in the 1910s, the primary concern was still maritime for northern ‘tropical’ Queensland, where the Torres Strait Islands proved a point of vulnerability ‘forming a chain of communication between Queensland and…the island extensions of Asia’.^80^ This region did not have the ‘geographical isolation’ afforded to the rest of the country and in fact, he believed the ‘limitations of maritime quarantine’ as designed by the Commonwealth for an island nation was exposed in this region.^81^ The ‘danger of cases of smallpox landing in the incubation period’ was temporally and geographically uneven across the country.^82^

The solution to address this inadequacy was clear to Elkington: vaccination. His experiences in resolving the 1903 smallpox epidemic in Launceston, Tasmania, confirmed his conviction of the benefits of a strict campaign, despite being unsuccessful in implementing lasting vaccination orders in that state.^83^ Indeed, even before his arrival there on 7 August 1903 as a specially appointed expert, he vocally ‘advocated wholesale vaccination’ to the press as the solution to the panic in Launceston.^84^ He took this conviction to his work in Queensland, noting in 1911 ‘the unvaccinated condition’ of the population ‘will render its [smallpox’s] suppression an undertaking of supreme difficulty’ should the virus be conveyed on shore.^85^ He put quarantine and vaccination together, the first line of defence a federal power, the second a state power, within his own capacity to devise and implement. Since ‘quarantine alone is wholly unable to prevent with certainty the entry’ of smallpox, Elkington concluded ‘the only real defence…is to be found in the organisation and powers provided by the State. In respect of smallpox this implies compulsory and closely enforced laws for vaccination and revaccination’.^86^ As mentioned, the *Health Act (1900)* in Queensland contained provisions for compulsory vaccination; however, these were never proclaimed. Aware of a common popular and sometimes expert caution over compulsion, Elkington was not ‘prepared to recommend the universal application of such statutory powers securing vaccination’ because his experience delivering public health in Tasmania and Victoria had shown him ‘[p]ublic opinion cannot be driven’ on this matter. He lamented a ‘widespread inability exists’ in Australia to recognise the true threat of smallpox and the virtues of vaccinations ‘to make the correct deductions’ towards preventative action.^87^

Cognizant of his inability to compel white Queenslanders to vaccinate and then revaccinate, he focused on a sub-population: Torres Strait Islanders. ‘So far as could be learned’ in his 1911 findings for Appel, Elkington reported that the Torres Strait Islands ‘were totally unprotected by vaccination’ presenting ‘the danger of introduction to Queensland through Thursday Island’.^88^ The threat from other infectious diseases was also articulated, demonstrating the pressing need, in his opinion, to conduct an inquiry ‘on the spot into the conditions’ of the islands as to ‘their possible effect on introduced epidemic disease’.^89^ He would then be able to develop ‘precise preventative measures applicable to the peculiar local conditions…save in the case of smallpox’.^90^ ‘It would be advantageous’, he advocated, ‘to combine with the inquiry a vaccination campaign designed to secure, if possible, the vaccinal protection of the entire population of each of the principal islands’, given the imminent threat of smallpox.^91^ This sanitary inspection tour combined with vaccination drive, was labelled by the *Brisbane Courier* a ‘frontier defence’ for Queensland.^92^

Having received the support of Home Secretary Appel, Protector of Aborigines Richard Baron Howard, and Dr Anton Breinl, the first director of the Australian Institute of Tropical Medicine (AITM) at Townsville, Elkington embarked on a three-month tour of the Torres Strait Islands and the Philippines on 5 April and returned to Brisbane on 25 June.^93^ The purpose of extending his medical survey into the Philippines, which was at the cost of the United States Government, was to study the ‘danger of infection from this quarter’ of the world.^94^ Or as one Queensland paper understood it, he went to the Philippines to learn about ‘the diseases likely to invade the northern part of Queensland’, acutely aware of the region’s heightened vulnerability.^95^

**Figure 2.**
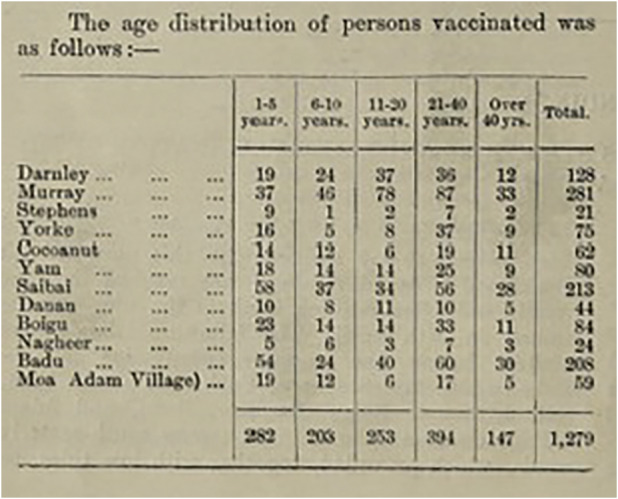
Table of vaccinated persons by age. Source: Queensland Department of Public Health, *Annual Report of the Commissioner of Public Health to 30th June 1912* (Brisbane: Anthony James Cumming, 1912), 22. Wellcome Collection.

It was in the first seven weeks of this tour that 1279 ‘natives…representing some 71 per cent’ of the Torres Strait Islanders were vaccinated against smallpox.^96^ Elkington was accompanied by his wife, health inspector Mr. C. M. Cato, and Mr. Frank Taylor, an entomologist from the AITM.^97^ While medical tours and even vaccination were routine work for health inspectors, this tour was considered of such ‘great importance’ that ‘the initial survey was made by the Commissioner for Public Health himself’.^98^ Indeed Elkington personally administered the vaccinations, according to individual reports written by the government teachers from the main islands.^99^ Figure 2 documents the population and number of vaccinations performed during the tour, which was pronounced a successful campaign, where ‘a “few bad arms” doubtless occurred’, but every operation was successful and there were no other reactions of note amongst the population.^100^ However, the low proportion of vaccinations on Murray Island was ‘hampered…by the uncontrolled enthusiasm displayed in dinghy racing’.^101^ While this shows that the vaccinations were not violently enforced upon Torres Strait Islanders, it remains the case that this demographic was targeted after acknowledging an inability to compel white Australians to vaccinate; thus the question of coercion is not one about physical force, but about when choice is offered. Elkington did not visit Mabuiag Island, which recorded a population of 250 Indigenous people that year, but the report stated that ‘Dr Wassell paid us a visit on 14 December, to vaccinate the natives, but the council met and asked him to postpone this until after the wet season, which he consented to do’.^102^ Whether Wassell did so or not, the following years are not in the records. However, vaccinations of Aboriginal and white people across Australia increased significantly in 1913 due to the largest outbreak of smallpox in Sydney that year. This surge in vaccinations, as already mentioned, particularly in the eastern states, lasted for two years after the epidemic, and perhaps because of this, targeted demographic vaccination programs were not an immediate priority for health administrators like Elkington in the following decade.

During his inspection tour, a case of smallpox was reported on 30 May 1912 aboard the *Yawata Maru*, which had landed at Thursday Island before the disease had been discovered.^103^ Around the same time, a case of plague was confirmed in Darwin, ‘the other Northern outpost’.^104^ Responding to these two threats, the Australian press praised Elkington calling his vaccination ‘precautions justified’ for the program’s ‘protection to the remainder of Australia’ was carried out ‘in the nick of time’.^105^ Eleven of the thirteen passengers who had disembarked at Thursday Island, ‘to make matters worse’, were pearlers who had already proceeded onto other vessels.^106^ The Acting Commissioner, Dr James Booth-Clarkson, pointed out the unmonitored surveillance on the movements of these pearlers as a concern because ‘if any cases were in the incubatory period, and the islanders had not been vaccinated, the disease might easily be distributed throughout Australia’.^107^ Affirming Booth-Clarkson’s appraisal of the situation, the *Brisbane Courier* reiterated that the ‘vaccination work performed has a very practical interest for Queensland, as it has broken the direct chain of communication which previously existed between New Guinea and Northern Queensland, in respect of the introduction of smallpox’.^108^ These two incidences of confirmed smallpox and plague threw into the public spotlight just how precarious Thursday Island was in the minds of health officials as that ‘back-door of Australia’ to Asia.^109^ Yet, as long as there was the ‘wisdom’ of policy and in people like Elkington, ‘the paramount importance’ of the Torres Strait Islands was also as a gate where ‘Asiatic diseases must be checked’ and where a program of targeted vaccinations provided a ‘belt of immunity from disease invasion’.^110^ Thus, the vaccination of these Indigenous people was not to bring hygienic practices to northern Queensland, but a mode of border protection for the state, conceived by one man. Elkington enacted within his powers to protect the state from a real though low risk of a smallpox outbreak. He was reluctant to enforce mass vaccination amongst the wider (white) population, but he was able to enmesh smallpox vaccinations with a colonial sanitation imperative, delivering, as he saw it, a much-needed immunity to the Torres Strait Islands.

The vulnerability that the Torres Strait posed on the rest of Australia’s health and the ‘good’ work of this vaccination drive was not quickly forgotten. In introducing the Torres Strait and its inhabitants to readers in 1929, Elkington’s program was recalled as one of civilising triumph, bringing medicine and care to ‘transform’ the Indigenous people into ‘contented, useful, and law-abiding people’.^111^ Their initial hesitation towards receiving vaccinations was recounted as amusing folly, but readers were reminded that this episode occurred in ‘our Northern gate’, that vulnerable coastline facing Asia.^112^

## 1933: Indigenous vaccination and federal administration

One generation later, a smaller but similar scheme was implemented in northern Australia by federal authorities. Like Elkington’s ideas, quarantine/vaccination was comprehended as a dual line of defense, and located deliberately on coastal First Nations communities deemed to be at once most vulnerable and most dangerous due to their proximity to South East Asia. In Elkington’s instance, this was simply about the proximity of Torres Strait Islanders under Australian jurisdiction to other Torres Strait islands and Islanders and to the Malay maritime world of traders, so closely connected itself to the Chinese market (and therefore, it was thought, to smallpox). In 1933, the point of contact was more intimate: the cosmopolitan crossover within the pearling industry in Darwin, capital of the Northern Territory, then administered not by a state government, like Queensland, but by the federal Commonwealth of Australia. Its health was thus under the portfolio of J.H.L. Cumpston.

**Figure 3.**
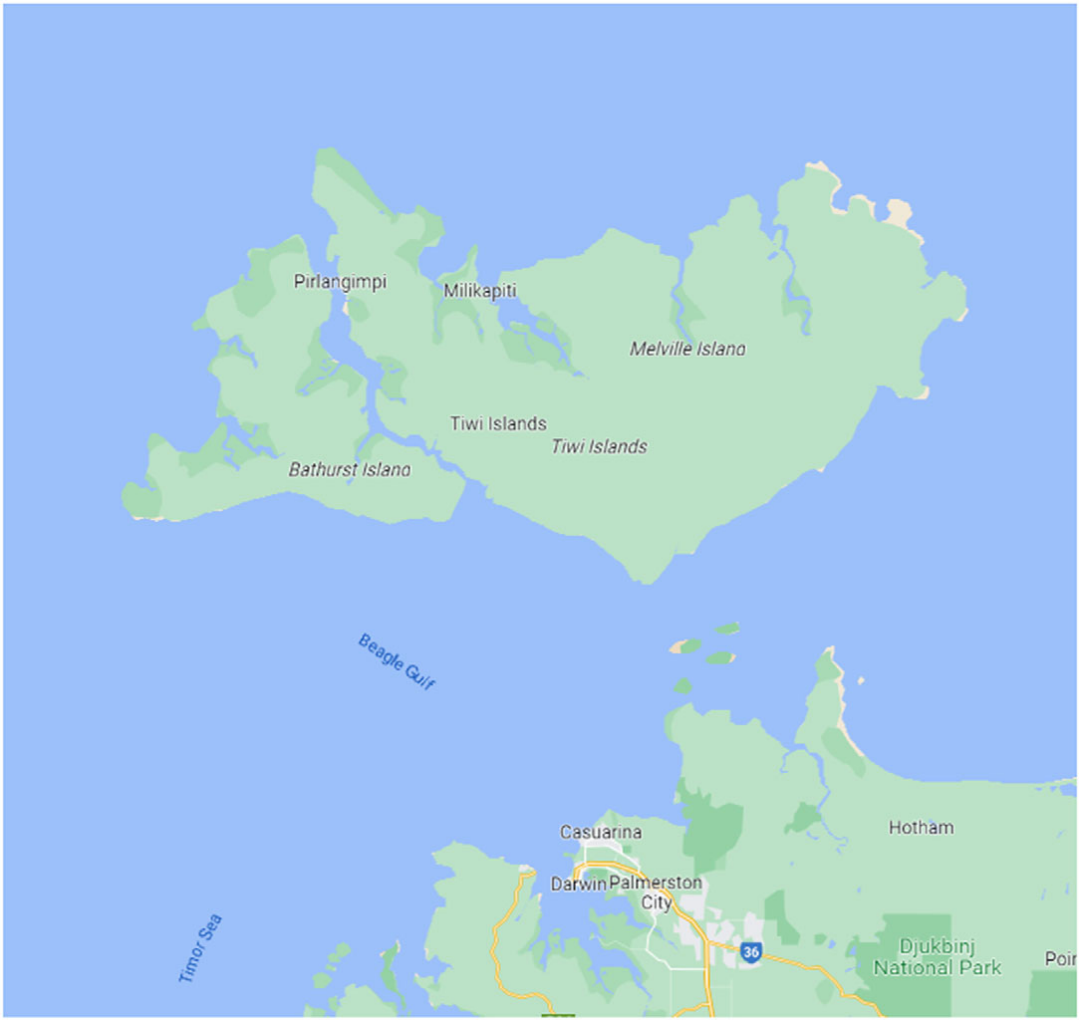
Map showing Darwin, Bathurst and Melville Island Source: Google Maps, 2023. *Darwin and Tiwi Islands.*

The *Report on the Administration of the Northern Territory* ending 30 June 1933 recorded the successful vaccination against smallpox of 212 Indigenous people on Bathurst Island and sixty more at Darwin (Figure 3). It was deemed necessary to vaccinate this population because authorities had that year identified the ‘risk of introduction of smallpox by the crew of foreign pearl-fishing vessels’.^113^ These crews—non-white indentured Japanese and Malay labourers—provided the workforce for this important but waning industry based off the coast of northern and north-west Australia.^114^ The ‘coastal aboriginals’ were selected for vaccination because they would ‘likely come into contact’ with the pearlers, into whose medical histories and vaccination status the Australian federal government had incomplete insight or no knowledge at all. With such a measure, a line of hygienic defense was created in and around the port of Darwin, allowing the administrators for the Northern Territory to report that ‘the health of the population generally has been good’ in the past twelve months, largely due to ‘the absence of any extensive outbreak of epidemic disease’.^115^ This result —no outbreak of smallpox—justified an additional year’s vaccination campaign, with a further 112 Indigenous people vaccinated from 1933 to 1934.^116^ This ‘policy of immunising the coastal aboriginal population against smallpox’ was the first time the Commonwealth Department of Health had implemented a targeted program similar to that authorised by a state government: the Queensland campaign implemented by Elkington.

How did this come about? On 23 March 1933, a lettergram was sent from Darwin to the Department of Interior in Canberra. It stated that the Chief Medical Officer and Protector of Aborigines, Dr Cecil Evelyn Cook, had conferred with the Director-General of Health, Dr J.H.L Cumpston, and they identified a risk of smallpox introduction by ‘alien pearlshell poachers in our waters’. These two medical men decided to ‘vaccinate all aboriginals [on] Melville and Bathurst Islands and along [the] north coast’.^117^ Cook had universal ambitions, but he was also realistic, acknowledging that his immunisation initiative might take ‘months or even years’ before he would ‘definitely state that every aboriginal along the coast has been vaccinated’.^118^ The intention was to vaccinate ‘a large proportion of the coastal population that the risk of introduction and dissemination of smallpox will be very considerably reduced this season’.^119^ He believed this task to be achievable and asked for ‘not less than 2,000 doses’ for that current season. Cook prioritised this work as the ‘first duties of the special Medical Officer’, a newly created position to oversee the medical service to coastal populations.^120^ It was estimated that there were 19,424 Indigenous people living in the Northern Territory in 1933, of whom 3,433 were in Darwin.^121^ J.W. Bleakley’s report in 1928 counted 150, 153, and 290 Indigenous people who were under permanent care at the Bathurst Island, Goulburn Island, and Millingimbi missions, respectively.^122^ From these numbers, problematic in their own ways, there were roughly 4,026 ‘coastal aboriginals’ in 1933. This means that around eleven per cent of the targeted population were vaccinated from 1933–34; hardly a ‘large proportion’. The ability to provide even a limited immunity against this disease speaks to the gravity of the smallpox concern, particularly heightened in this northern arena of mixed racial contact and unregulated entries into Australia.

Indeed, Cook and Cumpston located the risk of smallpox on the bodies of the ‘coloured’ pearl labourers, particularly the ‘alien’ pearlers, considered to be illegally operating off the shores of Darwin. They desired that ‘all coloured indents on pearling vessels from Darwin be subjected to vaccination since they associate with employers on foreign boats at fishing grounds’.^123^ Cook had consulted Mr Wolfensberger, the Sub-collector of Customs about the feasibility of vaccinations but had been advised thus: ‘There is apparently no power under the Immigration Act to require an indent to submit to vaccination.’^124^ However, Wolfensberger raised the possibility that the Minister for the Interior, John Perkins, ‘may be able to direct that indents desiring to remain in Australia should submit to vaccination’.^125^ Receiving ‘early finality’ to this question was pressing for Cook because it was ‘anticipated that all boats will shortly now be putting to sea’ to commence pearling.^126^ The response from the federal Department of the Interior cited precedent that it was ‘not desirable’ in their opinion to ‘subject those [labourers] at Darwin to treatment not enforced [in] other’ pearl shelling ports. However, the Commonwealth ‘appreciated circumstances [in] Darwin [were] different from other pearling centres’ and asked to have the full particulars forwarded to them for consideration.^127^

What circumstances were explicitly different in Darwin compared to other pearling ports in Australia is not explicitly stated, but there are two considerations. First, the industry off this coastline had expanded significantly in 1925 with the discovery of additional, rich pearl beds.^128^ These beds attracted an exodus of master pearlers, particularly from Broome to Darwin from 1928 onwards, bringing with them Malay and Japanese labourers in subsequent years.^129^ Second, the pearling industry in Darwin was solely under federal control, unlike the other two state-governed pearling towns, meaning that labour laws were not just different but were as yet undefined. As J.S. Bach notes, the Northern Territory was more economically favourable for master pearlers in the 1920s because the federal government was in a state of confusion and flux on how to regulate the remuneration and working conditions for Asian indentured labourers in this region’s industry.^130^ While the Immigration Act required indentured labourers to ‘be under contract or agreement to serve as part of the crew of a vessel’, it was unclear whether these labourers were governed by Western Australian labour agreements or by the federal Navigation Act.^131^ Seeking clarification, the Northern Territory government consulted local pearlers in 1929 and created a new pearling ordinance for the Territory that came into effect in 1931. This ordinance allowed the Minister to adjust the levels of remuneration dependent on the needs of the industry, but importantly for this paper, the ordinance obscured the provision of health and compensation to these labourers.

However, coloured labourers working in Darwin were still required to register with the federally appointed Sub-Collector of Customs on three-year contracts and have an identity card with a medical certificate. This allowed them to remain on-shore and work in menial jobs during the ‘lay-up season’ of three months. However, from a public health point of view, the intersecting federal laws created for this territory and this industry did not enable surveillance on or provision of medical well-being for these foreign labourers. As expressed by Cook and Cumpston, ‘these persons are those principally concerned in consulting with the crews of overseas vessels’ whose ‘neglect of the precaution’ of prophylaxis was a vulnerable point in Australia’s hygienic border.^132^ How could they monitor contact during those three months of the year when those labourers’ mobility was not tied to one vessel? This was not possible, and the vaccination of Aboriginal people was the alternative: a hygienic buffer in this zone of contact and irregular movement between Australia and the Malay world.

## Conclusion

As with other settler-colonial polities, Indigenous history in Australia was, and is, necessarily an element of national history, the founding element. Uniquely, however, in Australian history (and the history of the Pacific British Empire), there is also a foundational link between smallpox and colonisation, the much-discussed and researched introduction of the virus by the British (or possibly the French) in 1788, and the high mortality that followed almost immediately amongst Gadigal and Dharawal First Nations in and around present-day Sydney. An alternative thesis, which argues for the earlier introduction of smallpox through the coastal northern links with South East Asia and the Malay world, mirrors current historiographical ambitions to disturb the conventional geography of Australian national history: the desire to undo an over-privileged historical ‘origin’ with the British Empire.^133^

This article argues for an enduring significance of smallpox geographies in this settler colonial context. The two vaccination campaigns examined here introduce a health dimension to new historiographies about Australia’s coastal north and its connections to Southeast Asia. National medical histories are always regional medical histories, too. It pulls the long history of Indigenous people, smallpox, and government into the early twentieth century, into a period of intense nation-building that was unusually strongly connected to questions of health and infectious disease and to prevention campaigns that were themselves deeply invested in disease-free ‘white’ Australia. In focusing here on vaccination campaigns, the longstanding medical history interest in quarantine of the island-nation is complicated. These were two lines of defence. Yet the coastline being ‘defended’ in northern Australia was particular and one in which we can see the regulation of labour, immigration, and public health folding together, each a characteristic responsibility of modern government, each key in a high moment of nation-building, and each racialised in mutually constitutive ways.

The vaccination of *Indigenous* people of the coastlines, waters, and islands of northern Australia and the Torres Strait was imagined by public health personnel to be implemented in a frontier zone, but we have shown here not a history of marginality but of centrality, and not just in the obvious if important sense that this geography, this Country, was home to those vaccinated. The vaccination campaigns were in part an attempt to regulate the polyethnic relationships, populations, and connections that were idiosyncratically (health officials then would say problematically) part of northern coastal labour forces, communities, and economies. These two campaigns to vaccinate Indigenous people show that the process of bounding the island nation was uneven across geographies, shedding light on how borders in the north were hardened and gradually drawn into the centre of the national imaginary through public health.

## Footnotes

1
*The Simpsons*, 1995. Season 6, Episode 16, ‘Bart vs. Australia’, Directed by Wes Archer, Aired February 19; ‘Johnny Depp’s dogs face death in Australia’, *BBC*, 14 May 2015, https://www.bbc.com/news/world-australia-32732313.

2 ‘Fortress Australia’, *Herald Sun*, 20 March 2020; ‘The Covid-19 exit strategy: when will Australia know the coronavirus battle is over?’, *Guardian*, 11 April 2020; ‘Fortress Australia as PM shuts the gate’, *Sydney Morning Herald*, 20 May 2020; ‘Australia is the new hermit kingdom’, *Foreign Policy*, 11 May 2021; Lowy Institute, *Beyond Fortress Australia*, September 2021.

3 ‘Australia is the new hermit kingdom’, *Foreign Policy*, 11 May 2021.

4

Regina Jefferies , Jane McAdam , Sangeetha Pillai , ‘Can we still call Australia home? The right to return and the legality of Australia’s COVID-19 travel restrictions’, Australian Journal of Human Rights, 27, 2 (2021), 211–31.

5 ‘Australia is the New Hermit Kingdom’, *Foreign Policy*, 11 May 2021.

6 Commonwealth of Australia, Department of Health and Aged Care, ‘Arm yourself against COVID-19’, 11 July 2021, health.gov.au/news/arm-yourself-against-covid-19.

7 J.H.L Cumpston, ‘The protection of our frontiers from invasion by disease’, *Australasian Medical Gazette,* 20 July 1909, 347.

8 Dr Charles K. Mackellar’s, ‘Health Officer for the Government of New South Wales’ speech to the Royal Society of New South Wales in July 1883 is considered the start of a concerted push towards establishing federal quarantine. See: Charles K. MacKellar, *Federal Quarantine* (Sydney: Thomas Richards, 1883). The Australasian Sanitary Conference was subsequently held in Sydney, 1884, and the Australasian Quarantine Conference, Melbourne in 1896.

9

Alison Bashford , ‘Quarantine and the imaging of the Australian nation’, Health, 2, 4 (1998), 388 .

10 Cumpston, *op.cit.* (note 7), 347.

11 Of the existing literature, Judy Campbell’s monograph gives an overview of the sporadic and individual attempts to administer vaccinations in Indigenous communities. Michael Bennett places Australia in the early nineteenth-century global spread of vaccination technology and material, while Rebecca McWhirter focuses on the Australian colonies along the East Coast, and Alison Bashford takes a conceptual approach to the technology’s history. See: Judy Campbell, *Invisible Invaders: Smallpox and Other Diseases in Aboriginal Australia, 1780–1880* (Carlton: Melbourne University Press, 2002); Michael Bennett, ‘Smallpox and cowpox under the southern cross: The smallpox epidemic of 1789 and the advent of vaccination in colonial Australia’, *Bulletin of the History of Medicine,* 83, 1 (2009), 37–62; Rebecca McWhirter, ‘Lymph or liberty: responses to smallpox vaccination in the eastern Australian colonies’ (unpublished PhD thesis: University of Tasmania, 2008); Alison Bashford, *Imperial Hygiene: A Critical History of Colonialism, National and Public Health* (Basingstoke, Hampshire: Palgrave Macmillan, 2004), 14–38.

12 Office of the Commissioner of Public Health, *Annual Report of the Commissioner of Public Health to 30th June, 1912* (Brisbane: Anthony James Cumming, 1912); *Annual Report for the Chief Protector of Aboriginals for the Year 1912* (Brisbane: Anthony James Cumming, 1913).

13 ‘Letter from Weddell to Department of Interior’, 5 July 1933, National Archives of Australia, (hereafter NAA), A1 1933/2705.

14 For a critical overview of frontier historiographies in Australia see: Ben Silverstein, ‘Theoretical frontiers’, in Ann McGrath and Lynette Russell (eds), *The Routledge Companion to Global Indigenous History* (London: Routledge, 2021), 56–85.

15 Julia Martinez, ‘The end of indenture? Asian workers in the Australian pearling industry, 1901–1972’, *International Labor and Working-Class History,* 67 (2005), 125–47.

16

Regina Ganter , ‘Turing the map upside down’, History Compass, 4, 1 (2006), 26–35 .

17 A current ARC Laureate Fellowship project investigating Australia’s Indigenous peoples exploration and connections across the seas is Lynette Russell, *Global Encounters & First Nations Peoples: 1000 Years of Australian History*, https://www.monash.edu/arts/monash-indigenous-studies/global-encounters-and-first-nations-peoples. Existing scholarship see: Chris Urwin, Lynette Russell, Lily Yulianti, ‘Cross-cultural interaction across the Arafura and Timor Seas: Aboriginal people and Macassans in northern Australia’, in I.J. McNiven and B. David (eds), *The Oxford Handbook of the Archaeology of Indigenous Australia and New Guinea*, (Oxford: Oxford University Press, 2023); David Walker, ‘Perilous encounters: Australia, Asia and the Middle East’, in F. Mansouri (ed.), *Australia and the Middle East: A Front-line Relationship* (London, Tauris Academic Studies. 2006), 19–34; David Walker, *Anxious Nation: Australia and the Rise of Asia, 1850–1939* (St. Lucia; Queensland, University of Queensland Press, 1999); Regina Ganter, *Mixed Relations: Asian-Aboriginal Contact in North Australia* (Crawley: Western Australia, University of Western Australia Press, 2006); Regina Ganter, *op. cit.* (note 16); Regina Ganter, ‘The View from the North’, in Martyn Lyons and Penny Russell (eds), *Australia’s History: Themes and Debates*, (Sydney: UNSW Press, 2005), 41–62; Regina Ganter, ‘The Wakayama triangle: Japanese heritage of North Australia’, *Journal of Australian Studies* 23, 61 (1999), 55–63; Martinez, *op. cit.* (note 15); Julia Martinez and Adrian Vickers, *The Pearl Frontier: Indonesian Labor and Indigenous Encounters in Australia’s Northern Trading Network* (Honolulu: University of Hawaii Press, 2016); Julia Martinez, Claire Lowrie, Frances Steel and Victoria Haskins, *Colonialism and Male Domestic Service across the Asia Pacific* (London: Bloomsbury Academic, 2019); Claire Lowrie, ‘White “men” and their Chinese “boys”’: Sexuality, masculinity and colonial power in Singapore and Darwin, 1880s–1930s’, *History Australia,* 10,1 (2013), 35–57; Rebecca H. Bilous, ‘“All mucked up”: Sharing Stories of Yolŋu-Macassan cultural heritage at Bawaka, north-east Arnhem Land’, *International Journal of Heritage Studies,* 21, 9 (2015), 905–18.

18 John Mc Corquondale, ‘The legal classification of race in Australia’, *Aboriginal History,* 10, 1 (1986), 7–24; Jeremy Beckett (ed.), *Past and Present: The Construction of Aboriginality*, (Canberra: Aboriginal Studies Press, 1988); Victoria Haskins, ‘ ‘A better chance’? – Sexual abuse and the apprenticeship of Aboriginals girls under the NSW Aborigines Protection Board’, *Aboriginal History*, 88, 147–64; Sylvia Kleinert, ‘Aboriginal enterprises: Negotiating an urban Aboriginality’, *Aboriginal History,* 23, 171–96.

19 This is explicitly argued in Ganter, *Mixed Relations*, *op. cit.* (note 17), 237.

20 Bashford, *op. cit.* (note 9); Alison Bashford, ‘At the border: Contagion, immigration, nation’, *Australian Historical Studies,* 120 (2002), 344–58; Frank Broeze, *Island Nation: A History of Australians and the Sea* (North Sydney: Allen & Unwin, 1998).

21 Bashford, *op. cit.* (note 9).

22

Alison Bashford , ‘“Is White Australia possible?”: Race, colonialism and tropical medicine in the early twentieth century’, Ethnic and Racial Studies, 23 (2000), 112–35; Warwick Anderson , The Cultivation of Whiteness: Science, Health, and Racial Identity in Australia (Melbourne: Melbourne University Press, 2002).

23

Claire Hooker , ‘Diphtheria, immunisation and the Bundaberg tragedy: A study of public health in Australia’, Health and History, 2, 1, 52–78 ; Peter Hobbins , ‘”Immunisation is as popular as a death adder”: The Bundaberg tragedy and the politics of medical science in interwar Australia’, Social History of Medicine, 24, 2, 426–44.

24 See: Paul Kelton , Cherokee Medicine, Colonial Germs: An Indigenous Nation’s Fight against Smallpox, 1518–1824 (Norman: University of Oklahoma Press, 2015); Seth Archer , ‘Colonialism and other afflications: Rethinking Native American health history’, History Compass, 14, 10 (2016): 511–1; Russel Viljoen, ‘Cape of contagion: Cape Town, contagion and the curse of smallpox (ca. 1713, 1755, and 1767)’ in Poonam Bala and Russel Viljoen (eds), *Epidemic Encounters, Communities, and Practices in the Colonial World* (Landham, MD: Lexington, 2023), 85–114.

25 Judy Campbell, *op. cit.* (note 11), 61 & 71–75; C.C. Macknight, ‘Macassans and the Aboriginal Past’, *Archaeology in Oceania* 21, 1 (1986), 69–75.

26 Noel Butlin, *Our Original Aggression: Aboriginal Populations of Southeastern Australia, 1788–1850*, (Sydney: Allen & Unwin, 1983); N.G. Butlin, ‘Macassans and Aboriginal Smallpox: The “1789” and “1829” epidemics’, *Australian Historical Studies,* 21, 84 (2008), 315–35; Craig Mear, ‘The origin of the smallpox outbreak in Sydney in 1789’, *Journal of the Royal Australian Historical Society*, 94, 1 (2008), 1+; Frank Fenner, ‘Smallpox, “the most dreadful scourge of the human species”: Its global spread and recent eradication’, *Medical Journal of Australia*, 141 (1984), 731; Bashford, *Imperial Hygiene*, *op. cit.* (note 9), 41; Michael J. Bennett, *op. cit.* (note 11).

27 Fenner, *op. cit.* (note 26), 731; Bashford, *Imperial Hygiene*, *op. cit.* (note 9), 41.

28 Campbell, *op.cit.* (note 11), 12–13.

29 J.H.L Cumpston, *Health and Disease in Australia: A History*, introduced and edited by M.J. Lewis, (Canberra: Australian Government Publishing Service, 1989), 186.

30 Anon, ‘History of Smallpox in Australia, 1909-1923’. *Public Health Reports (1896–1970),* 40, 33 (1925), 1689.

31 Office of the Commissioner of Public Health, *Annual Report of the Commissioner of Public Health to 30th June, 1912* (Brisbane: Anthony James Cumming, 1912), 5.

32
*Ibid.*

33

Francis Campbell , ‘Vaccination’, NSW Legislative Council Journal, 1, 66 (1867–8), 830 .

34

H.G. Alleyne , ‘Vaccination’, NSW Legislative Council Journal, 2 (1878–9), 931 .

35

Peter Curson , Times of Crisis: Epidemics in Sydney, 1788–1900, (Sydney: Sydney University Press, 1985).

36

Alison Bashford , ‘Epidemic and governmentality: Smallpox in Sydney, 1881’, Critical Public Health, 9, 4 (1999), 301–16.

37
*Ibid.*

38

Michael Roe , ‘Smallpox in Launceston, 1887 and 1903’, Paper and Proceedings: Tasmanian Historical Research Association, 23, 4 (1976), 111–48.

39

Krista Maglen , ‘A world apart: Geography, Australian quarantine, and the mother country’, Journal of the History of Medicine and Allied Sciences, 60, 2 (2005), 196–217 15737958 10.1093/jhmas/jri023.

40

Peter Hobbins , ‘‘Union Jack or Yellow Jack?’ Smallpox, sailors, settlers and sovereignty’, Journal of Imperial and Commonwealth Studies, 45, 3 (2017), 391–415 .

41
*Ibid.*, 405.

42 J.H.L. Cumpston, *The History of Smallpox in Australia, 1788–1908 (*Melbourne: Albert J. Mullett, Government Printer, 1914), 11.

43 ‘Small-pox in Sydney’, *Ballarat Courier*, 30 May 1881, 3.

44 For literature on racial vilification in the nineteenth and early twentieth centuries see: Alan M. Kraut, *Silent Travelers: Germs, Genes, and the ‘Immigrant Menace’ (*New York: Basic Books, 1994); Nayan Shah, *Contagious Divides: Epidemics and Race in San Fransisco’s Chinatown* (Berkeley: University of California Press, 2001); Guenter B. Risse, *Plague, Fear, and Politics in San Fransisco’s Chinatown* (Baltimore: Johns Hopkins University Press, 2012); Peter Curson and Kevin McMracken, *Plague in Sydney: An Anatomy of an Epidemic* (Sydney: New South Wales University Press, 1989), 174–6.

45

Daniel A. Salmon et al., ‘Compulsory vaccination and conscientious or philosophical exemptions: Past, present and future’, The Lancet, 367, 908, 4–10 10.1016/S0140-6736(06)68144-016458770.

46 Cumpston, *op. cit.* (note 42), 136.

47
*Ibid.*, 140.

48 ‘Case of smallpox, Precautions in Brisbane’, *The Telegraph*, 2 Sept 1911, 2; ‘The smallpox incident’, *Brisbane Courier*, 4 September 1911, 7; ‘Compulsory vaccination’, *Gympie Times and Mary River Mining Gazette*, 14 September 1911, 1.

49 Cumpston, *op. cit.* (note 42), 132.

50
*Ibid.*

51 J.H.L. Cumpston and F. McCallum, *The History of Small-pox in Australia 1901–1923* (Melbourne: H.J. Green, Government Printer, 1925), 109.

52 Cumpston, *op. cit.* (note 42), 133.

53 Cumpston, *op. cit.* (note 42), 133–4.

54 Anon, *op. cit.* (note 30), 1691.

55 ‘Domestic intelligence’, *Sydney Morning Herald*, 31 Oct 1831, 4; Cumpston, *op. cit.* (note 42), 150 and 153.

56 Campbell, *op. cit.* (note 11), 129.

57
*Ibid.*, 212.

58 ‘Report of Protector of Natives’, *Inquirer*, 18 February 1852, 3.

59 Campbell, *op. cit.* (note 11), 193.

60 ‘Report of Protector of Natives’, *Inquirer*, 18 February 1852, 3.

61 ‘Report of the Guardian of Aborigines’, *Perth Gazette and Independent Journal of Politics and News*, 27 January 1854, 3.

62 The intention to vaccinate the Indigenous population was registered, but it is unclear in the sources whether these vaccinations were carried out and to what extent. Cecil Cook, ‘Medicine and the Australian Aboriginal: A century of contact in Northern Territory’, *Medical Journal of Australia*, 1 (1966), 562.

63 ‘Westralia’, *Northern Western Advocate and the Emu Bay Times*, 25 July 1904, 2.

64 Office of the Commissioner of Public Health, *Annual Report of the Commissioner of Public Health* (Brisbane: Office of the Commissioner of Public Health, 1910), 5.

65 Office of the Commissioner of Public Health, *Annual Report of the Commissioner of Public Health* (Brisbane, Office of the Commissioner of Public Health, 1908), 11.

66 Office of the Commissioner of Public Health, *op. cit.* (note 64), 4.

67
*Ibid.*, 5.

68 ‘Aboriginal Protection’, *Cairns Post*, 19 April 1911, 5; ‘Care of Aborigines’, *Brisbane Courier*, 19 April 1911, 5; ‘Home Secretary’s Tour’, *Telegraph*, 24 April 1911, 7; ‘Visiting the Mission, Mr Appel’s Tour’, *Cairns Post,* 25 April 1911, 4.

69 ‘Keeping out Disease’, *Brisbane Courier*, 22 July 1911, 4.

70 ‘Danger of Epidemics’, *Northern Miner*, 7 August 1911, 3.

71
*Ibid.*

72 ‘Islands of Torres Straits the Home Secretary’s Visit Condition of the Natives’, *Brisbane Courier*, 24 May 1911, 6.

73 Office of the Commissioner of Public Health, *Annual Report of the Commissioner of Public Health to 30th June 1911* (Brisbane: Anthony James Cumming, 1911), 3.

74 ‘Danger of Epidemics’, *Northern Miner*, 7 August 1911, 3.

75 Office of the Commissioner of Public Health, *op. cit.* (note 73), 9; ‘Public Health’, *Telegraph*, 23 August 1911, 13.

76
*Ibid.*

77
*Ibid.*, 4.

78
*Ibid.*

79
*Ibid.*, 133–5.

80 ‘Danger of Epidemics’, *Northern Miner*, 7 August 1911, 3.

81 Office of the Commissioner of Public Health, *Annual Report of the Commissioner of Public Health to 30th June 1912* (Brisbane: Anthony Jame Cumming, 1912), 10.

82 Office of the Commissioner of Public Health, *op. cit.* (note 73), 9.

83 Murray Johnson, ‘The 1903 smallpox outbreak at Launceston and the role of J.S.C. Elkington’, *Launceston General Hospital Historical Committee: Papers and Proceedings,* (2005), 17–22.

84 ‘Smallpox’, *Sydney Morning Herald*, 8 August 1903, 9. Coverage of Elkington’s advice before arriving in the city include: ‘Stricken Launceston’, *Herald*, 6 August 1903, 4; ‘Smallpox at Launceston’, *Age*, 7 August 1903, 6; ‘Smallpox in Tasmania’, *Advertiser*, 7 August 1903,5; ‘Dr. Elkington’s Recommendations’, *Tasmanian News*, 7 August 1903, 2.

85 Office of the Commissioner of Public Health, *op. cit.* (note 73), 9.

86
*Ibid.*

87
*Ibid.*

88 ‘Danger of Epidemics’, *Northern Miner*, 7 August 1911, 3.

89
*Ibid.*

90 ‘Frontier Defence’, *Brisbane Courier,* 29 July 1911, 5; *Ibid.*

91 Danger of Epidemics’, *Northern Miner*, 7 August 1911, 3.

92 ‘Frontier Defence’, *Brisbane Courier*, 29 July 1811, 5.

93 ‘Untitled’, *Zeehan and Dundas Herald*, 3 April 1912, 2; ‘Personal’, *Daily Mercury,* 9 April 1912, 7; ‘Personal’, *Townsville Daily Bulletin*, 15 April 1912, 4; ‘Health Commissioner: Return from the East’, *Telegraph*, 26 June 1912, 4.

94 ‘Dr. Elkington’s Tour’, *Telegraph*, 16 May 1912.

95 ‘Queensland’, *Kalgoorlie Miner*, 17 May 1912, 8; ‘Public Health’, *Bundaberg Mail and Burnett Advertiser*, 17 May 1912, 2. ‘Queensland Health Inspector’s Mission’, Evening Journal, 17 May 1912, 1.

96 Office of the Commissioner of Public Health, *op. cit.* (note 81), 22.

97
*Annual Report of the Chief Protector of Aboriginals for the Year 1912* (Brisbane: Anthony James Cumming, 1913), 26; For information on Cato and Taylor see: ‘C.M. Cato Retiring’, *Telegraph*, 24 June 1953, 15; ‘Death of Mr F.H. Taylor’, *Sydney Morning Herald,* 28 December 1945, 5.

98 ‘Fighting Disease’, *Brisbane Courier*, 4 July 1912, 4.

99
*Op. cit.* (note 97), 21–7.

100 Office of the Commissioner of Public Health, *op. cit.* (note 81), 22.

101 Cumpston and McCallum, *op. cit.* (note 51), 105.

102
*Op. cit.* (note 97), 21–7.

103 ‘Smallpox and Plague’, *Brisbane Courier*, 30 May 1912, 7.

104 ‘The Northern Outpost’, *Darling Downs Gazette*, 1 June 1912, 4.

105 ‘Precautions Justified’, *Argus*, 30 May 1912, 8.

106
*Ibid.*

107 ‘Smallpox and Plague’, *Brisbane Courier,* 30 May 1912, 7.

108 ‘Fighting Disease’, *Brisbane Courier*, 4 July 1912, 4.

109 ‘The Northern Outpost’, *Darling Downs Gazette*, 1 June 1912, 4.

110 ‘Smallpox and Plague’, *Telegraph*, 30 May 1912, 2; ‘The Northern Outpost’, *Darling Downs Gazette*, 1 June 1912, 4; ‘Dr. Elkington’s Tour’, *Telegraph*, 16 May 1912; ‘Queensland Health Inspector’s Mission’, *Evening Journal*, 17 May 1912, 1; ‘Combating Disease, Vaccinating the Natives’, *Sydney Morning Herald*, 18 May 1912, 18.

111 ‘Torres Strait Islanders. Communal Life’, *Brisbane Courier*, 14 December 1929, 23.

112
*Ibid.*

113 Australia Parliament & Northern Territory Administration, *Report on the Administration of the Northern Territory*, 1932/33, 26.

114 Ganter, Mixed Relations, op. cit. (note 17), 66.

115 Australia Parliament & Northern Territory Administration, *op. cit.* (note 113), 26.

116 Australia Parliament & Northern Territory Administration, *Report on the Administration of the Northern Territory*, 1933/34, 29.

117 ‘Lettergram, Department Interior’, 23 March 1933, NAA A1 1933/2705.

118 ‘Letter from Cook to Weddell’, 25 March 1933, NAA: A1, 1933/2705.

119
*Ibid.*

120 ‘Letter from Cook to Weddell’, 22 March 1933, NAA: A1, 1933/2705.

121
*Report on the Administration of the Northern Territory for the Year ended 30^th^ June, 1933* (Canberra: L.F. Johnston, 1934), 10.

122 J.W. Bleakley, *The Aboriginals and Half-Castes of Central Australia and North Australia* (H.J. Green, 1929), 12.

123 ‘Letter from Cook to Weddell’, 22 March 1933, NAA: A1, 1933/2705.

124
*Ibid.*

125
*Ibid.*

126
*Ibid.*

127 ‘Letter from Brown to Cumpston’, 22 May 1933, NAA A1, 1933/2705.

128 J.P.S. Bach, *The Pearling Industry of Australia: An Account of its Social and Economic Development*, (Canberra: Commonwealth of Australia, 1965), 165.

129 Martinez and Vickers, *op. cit.* (note 17), 86–7; *Ibid.*, 167.

130 Bach, *op. cit.* (note 128), 166–9.

131 Australia*, Immigration Restriction Act 1901*, 3(g).

132 ‘Letter from Cook to Weddell’, 22 March 1933, NAA A1, 1933/2705.

133 Alison Bashford and Stuart Macintyre, ‘Introduction’, *The Cambridge History of Australia: vol 1 Indigenous and Colonial Australia* (Melbourne: Cambridge University Press, 2013), 1–13.

